# Balancing Early Detection and Biopsy Risks in Sub-centimeter Lung Nodules: A Case Series

**DOI:** 10.7759/cureus.101186

**Published:** 2026-01-09

**Authors:** Joseph O Odeyemi, Leah Whitis, Reem Mumtaz, Abdel-Ghanie Abu-Samra

**Affiliations:** 1 Internal Medicine/Pediatrics, University of Kansas School of Medicine, Wichita, USA; 2 Internal Medicine, University of Kansas School of Medicine Wichita, Wichita, USA; 3 Pulmonology/Critical Care, University of Kansas School of Medicine Wichita, Wichita, USA

**Keywords:** ct-guided biopsy, early cancer detection, lung cancer screening, pulmonary nodules, robotic bronchoscopy

## Abstract

Pulmonary nodules smaller than 8 mm are often monitored over time. In some patients, these nodules may grow while under observation, occasionally representing aggressive disease. We describe three patients with initially small pulmonary nodules that demonstrated significant growth during follow-up. Two were ultimately found to have small-cell lung carcinoma, and one had probable non-small cell carcinoma. These cases highlight the potential limitations of delayed tissue sampling and suggest that earlier diagnostic evaluation may be beneficial in certain patients. Advanced bronchoscopic techniques can allow safe and accurate biopsy of small, peripheral nodules. Earlier intervention in patients with growing nodules could support timely treatment and improve outcomes.

## Introduction

Pulmonary nodules are small (≤3 cm), well-defined lung lesions, surrounded by normal lung tissue, and usually not associated with other lung abnormalities, such as lymphadenopathy, pleural effusion, or atelectasis [1]. On the other hand, lung masses are >3 cm and can be associated with other radiological abnormalities [1]. Lung masses are more likely to be malignant than lung nodules [2].

Pulmonary nodules can be classified into solid, part-solid, or ground glass nodules based on their appearance on imaging [3]. The etiology for pulmonary nodules is extensive and ranges from infective and inflammatory causes to benign neoplasms and malignancy. Of note, >90% of all pulmonary nodules are benign [4]. The risk of malignancy is directly proportional to the size of the nodule, with nodules <5 mm with a <1% chance of malignancy, nodules 5-9 mm with a 2% to 6% probability, nodules 8-20 mm with an 18% chance, and nodules >20 mm with a >30% probability of malignancy [5,6].

However, factors other than nodule size predict malignancy. These include growing nodules, part-solid nodules, the presence of spiculated or irregular borders, advanced age, and the presence of lung cancer risk factors such as cigarette smoking, prior malignancy, chronic obstructive pulmonary disease (COPD), and asbestos exposure [2].

With the utilization of low-dose chest computed tomography (CT) scans for lung cancer screening and the overall increase in CT imaging, the diagnosis of pulmonary nodules has increased drastically [7]. In the National Lung Screening Trial (NLST), about 40% of participants meeting lung cancer screening criteria had a pulmonary nodule [8]. A similar study by the Veterans Health Administration found that up to 60% of those screened had lung nodules, with 13% having nodules >8 mm [8]. Among adults, the frequency of chest CT scans increased from around 1% to 2% between 2006 and 2012, with an increase in the incidental detection of lung nodules to about 30% during that period [7]. Some studies have evaluated the prevalence of clinically significant lung nodules. The frequency of nodules ≥4 mm has been reported as high as 25%, while nodules >6 mm have been found in up to 10% of cases [9].

For the management of pulmonary nodules, the guidelines aim to balance the benefits of early detection with the potential risks of unnecessary procedures, which is a clinical challenge in the management of sub-centimeter pulmonary nodules. The current guidelines recommend tissue sampling of high- or intermediate-risk nodules >8 mm; however, for nodules ≤8 mm, they generally recommend repeat imaging to assess nodule growth [3].

This paper presents three cases of high-risk patients with pulmonary nodules smaller than 8 mm, in which the nodules showed significant growth while awaiting repeat imaging. This raises the question of whether immediate tissue sampling should be recommended for high- or intermediate-risk small pulmonary nodules. Additionally, it prompts consideration of whether earlier biopsies of even smaller nodules could impact current management strategies by increasing the success rate of curative resections and reducing the need for chemoradiation, which carries significant adverse effects.

Specifically, for small-cell lung carcinoma (SCLC), surgery is generally not indicated due to its aggressive nature and early metastatic spread, resulting in a low (<5%) rate of stage I SCLC diagnosis [10]. This leads to the question: could earlier biopsies facilitate earlier diagnosis of SCLC, potentially altering the standard of care in the future?

One important advancement that has significantly shaped this discussion is the emergence of robotic-assisted bronchoscopy (RAB), such as the ION endoluminal system. RAB offers the potential to achieve high diagnostic accuracy with minimal trauma [11,12]. Studies have demonstrated that RAB yields diagnostic sensitivity and specificity comparable to that of CT-guided transthoracic biopsy and superior to traditional transbronchial biopsies, but with substantially lower complication rates than both methods [11-13]. Notably, RAB appears to offer a particular advantage in evaluating smaller pulmonary nodules and lesions located in anatomically challenging regions of the lung [12,14,15].

## Case presentation

Case 1

**Figure 1:**
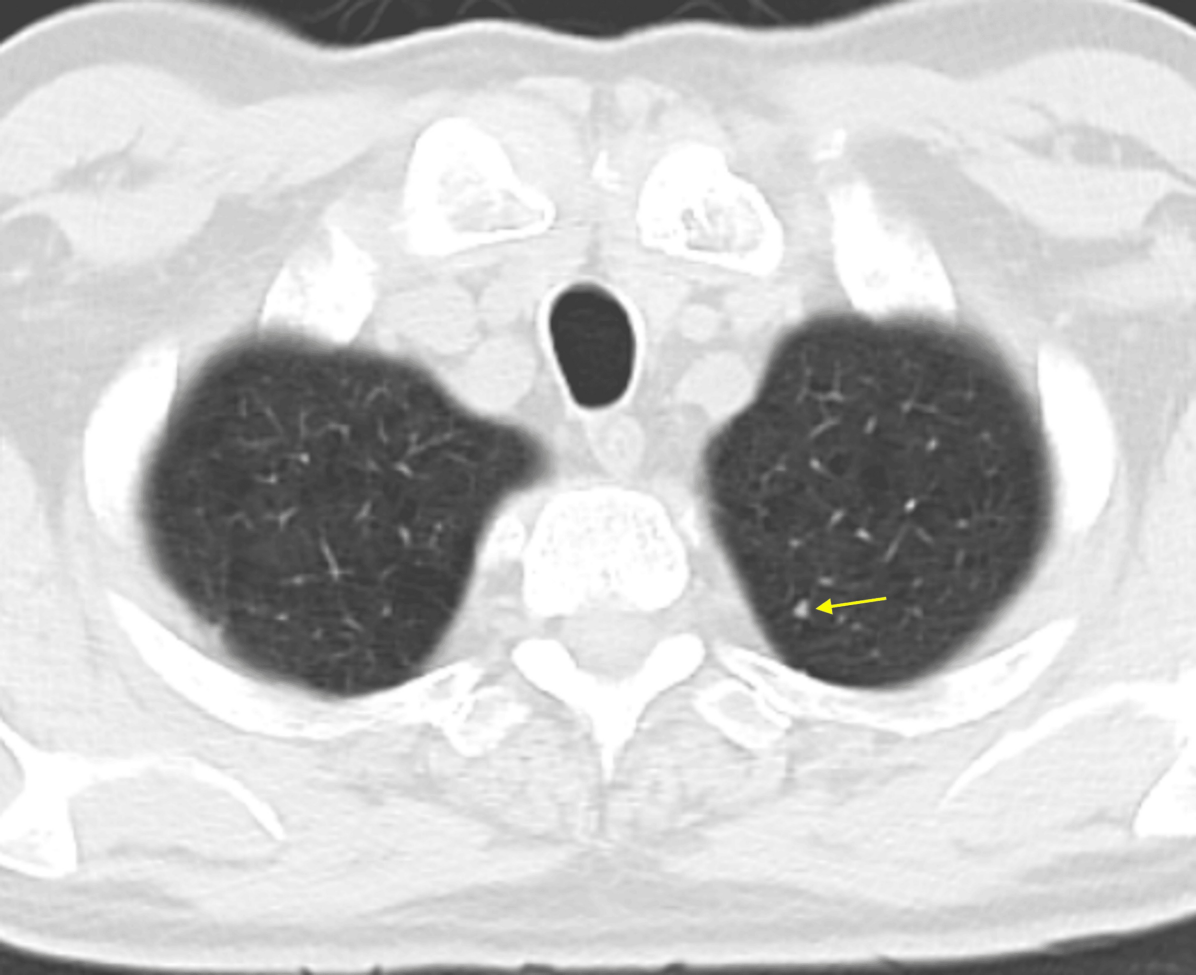
Chest CT scan in October 2022 showing a small 3-4 mm nodule in the upper lobe of the left lung CT: computed tomography

The patient is a 66-year-old male with a history of tobacco dependence (60 pack-year smoking history), moderately severe COPD with upper lobe predominant emphysema, hypertension, atrial fibrillation, and type 2 diabetes mellitus. In October 2022, a screening chest CT scan revealed a small 3-4 mm nodule in the left lung apex (Figure 1).

**Figure 2:**
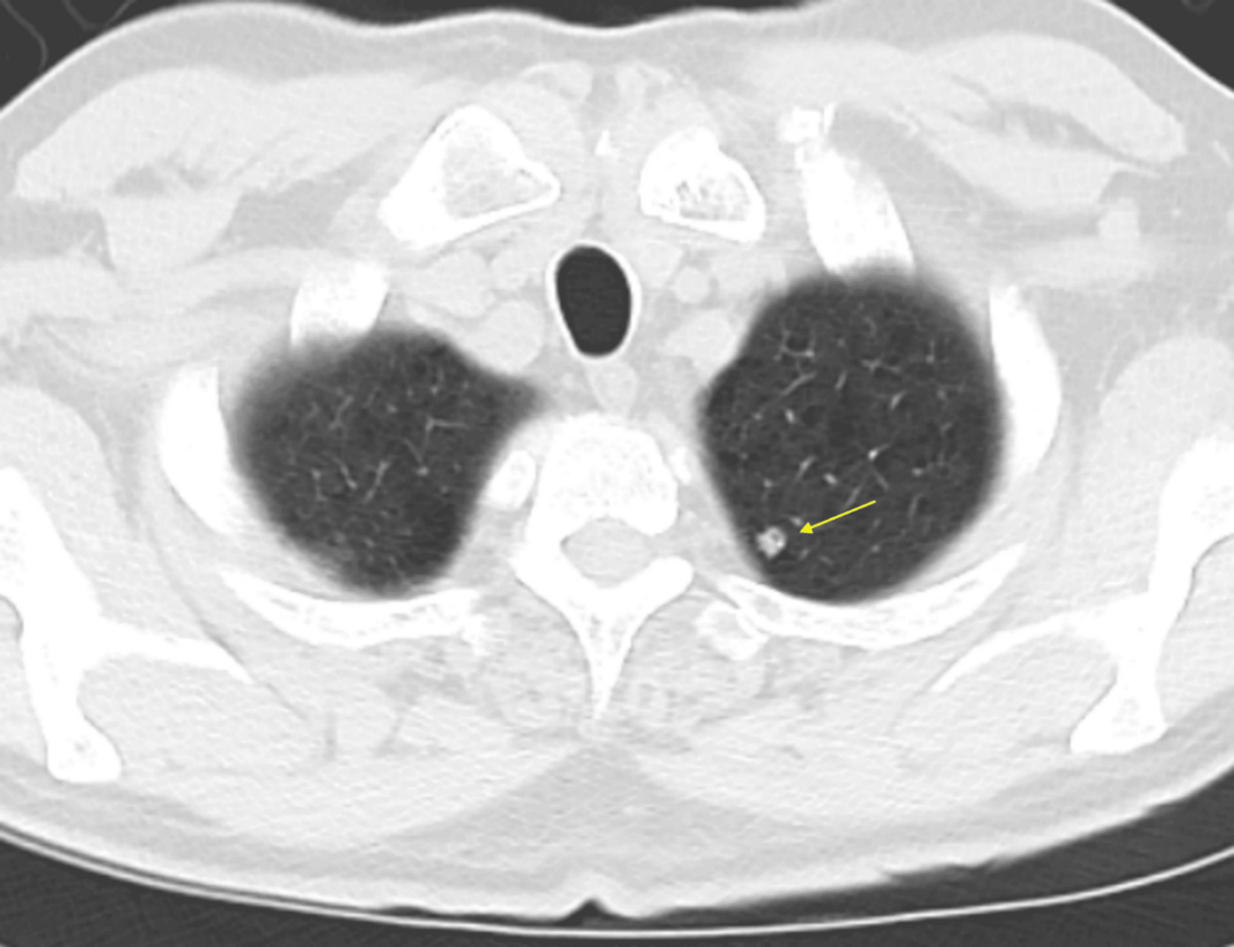
Chest CT scan in October 2023 showing a 7 x 5 mm nodule in the upper lobe of the left lung CT: computed tomography

Based on the Fleischner criteria, a plan was made to monitor the nodule with follow-up imaging. A repeat chest CT in October 2023 showed that the nodule had increased in size to 7 x 5 mm, and the recommendation was to follow it more closely with imaging in three months (Figure 2).

**Figure 3:**
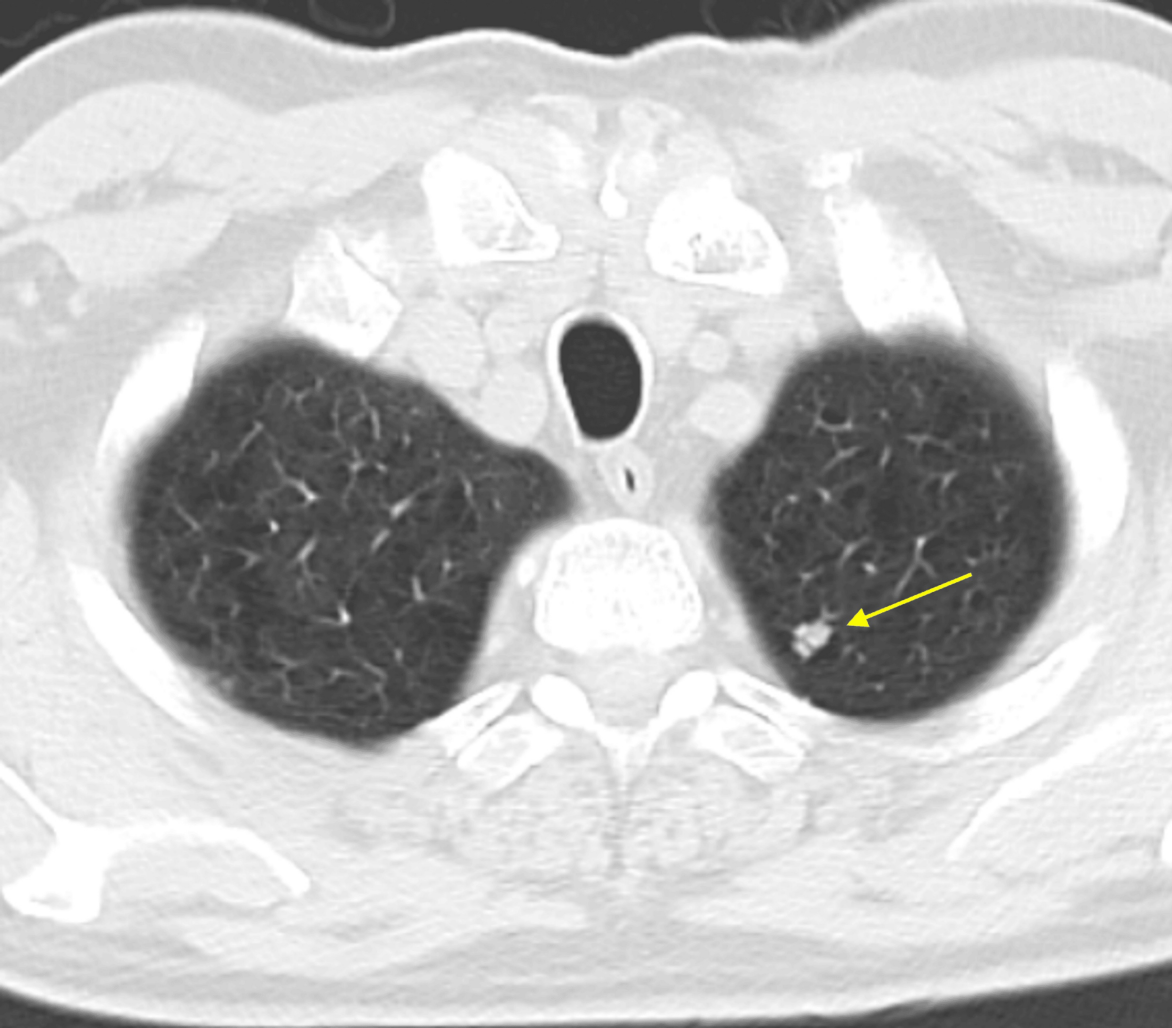
Chest CT scan in March 2024 showing a 8 x 6 mm nodule in the upper lobe of the left lung CT: computed tomography

A chest CT was repeated in March 2024, five months later, showing that the left upper lobe pulmonary nodule had grown to approximately 8 x 6 mm (Figure 3). There was no evidence of mediastinal or axillary lymphadenopathy meeting CT size criteria for adenopathy.

**Figure 4:**
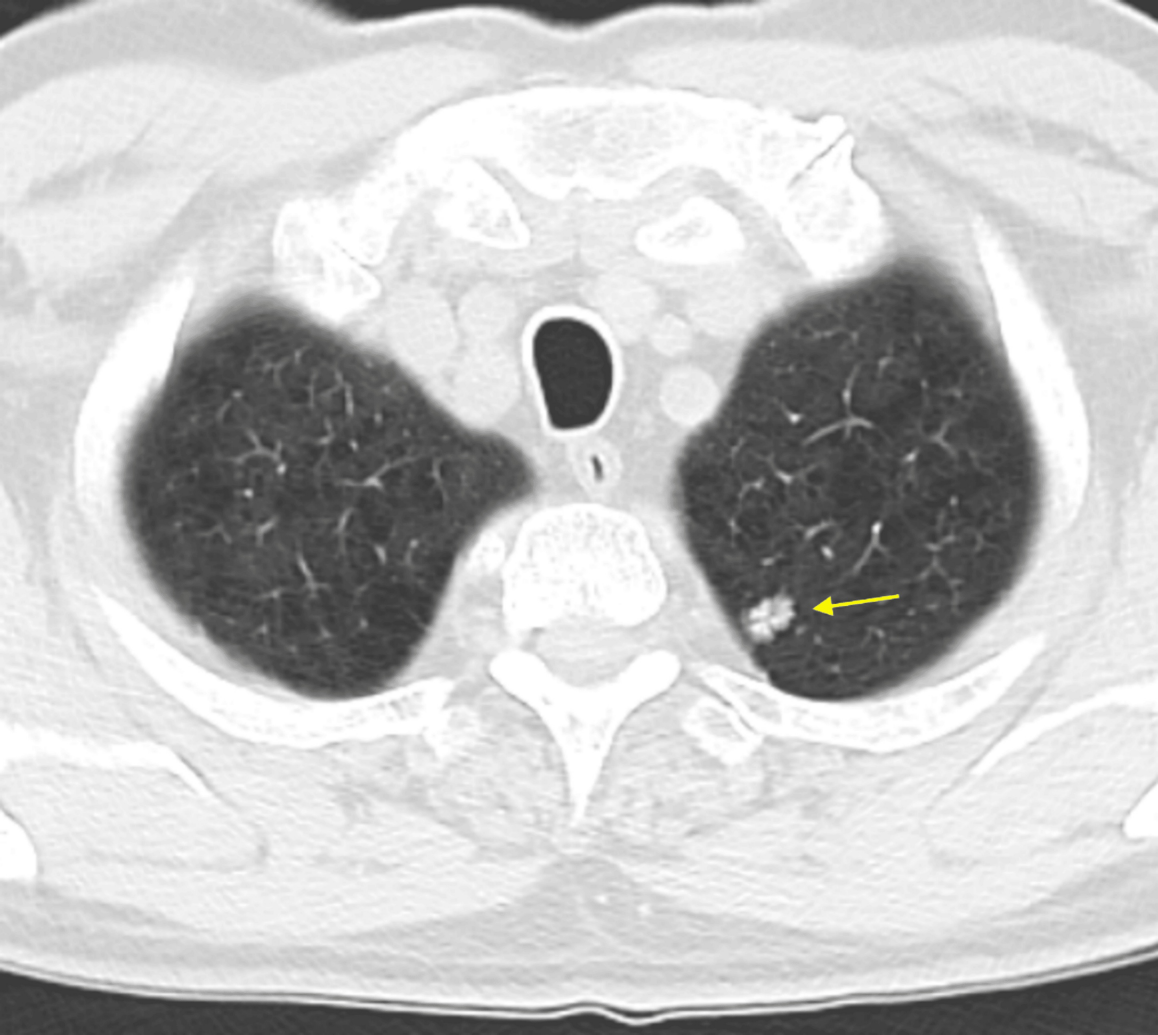
Chest CT scan in October 2024 showing a 11 x 8 mm nodule in the upper lobe of the left lung CT: computed tomography

CT-guided biopsy, ION RAB, and imaging surveillance were discussed with the patient. However, the patient was reluctant to undergo any interventions, stating that he preferred to wait and repeat imaging in six months. He expressed willingness to consider a biopsy if the lesion continued to grow during this period. A repeat chest CT scan in October 2024 showed continued growth of the left apical lung nodule, which now measured 11 x 8 mm and had eccentric calcifications (Figure 4). No associated lymphadenopathy was noted. A fluorine-18 fluorodeoxyglucose positron emission tomography (PET) scan in November 2024 showed a mildly hypermetabolic left upper lobe pulmonary nodule with a maximum standardized uptake value (SUV) of 2.7, raising concern for a pulmonary neoplasm (Image not available). There were no other hypermetabolic findings suggestive of metastatic disease.

**Figure 5:**
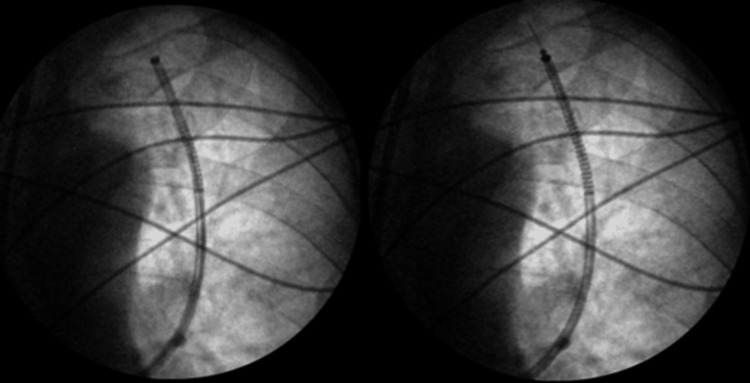
Fluoroscopic images obtained during ION RAB showing the catheter trajectory as it advanced toward the target lesion for biopsy RAB: robotic-assisted bronchoscopy

Subsequently, the patient was scheduled for an ION RAB with EBUS and transbronchial biopsy in November 2024 (Figure 5).

The pathology results were consistent with SCLC. Following this, the cardiothoracic surgery team was consulted, and the patient underwent a robotic left upper lobe segmentectomy with clear margins later that month. He was also referred to oncology for further management. The oncology team recommended adjuvant chemotherapy, but the patient declined and opted for active surveillance.

Case 2

**Figure 6:**
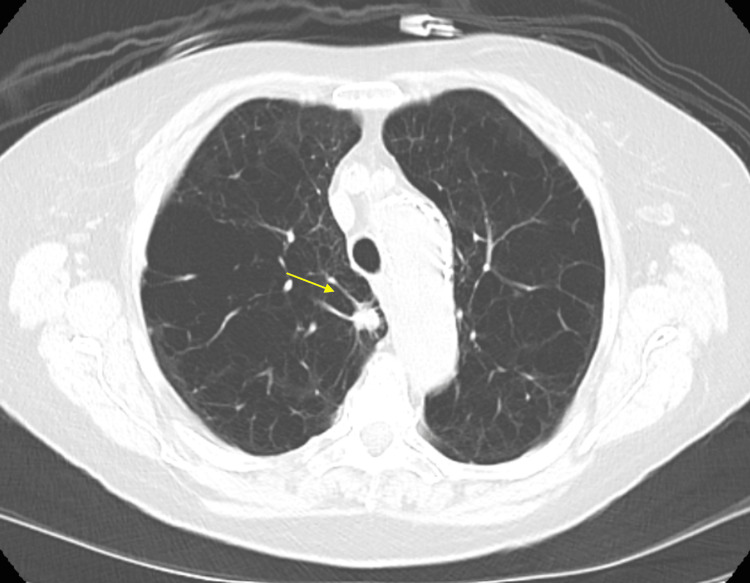
Chest CT scan in October 2024 showing a spiculated 13 x 12 mm nodule in the upper lobe of the right lung CT: computed tomography

The patient is a 78-year-old woman with a history of tobacco dependence and COPD, currently on home oxygen. She was initially diagnosed with a small right upper lobe nodule (<8 mm) identified on a screening chest CT scan in October 2023 (CT image not available). A repeat chest CT scan in October 2024 showed a spiculated right upper lobe nodule measuring approximately 1.3 x 1.2 cm, which had increased in size from the previous scan (Figure 6). Additional small pulmonary nodules in the upper lung zones were present but stable since 2023.

**Figure 7:**
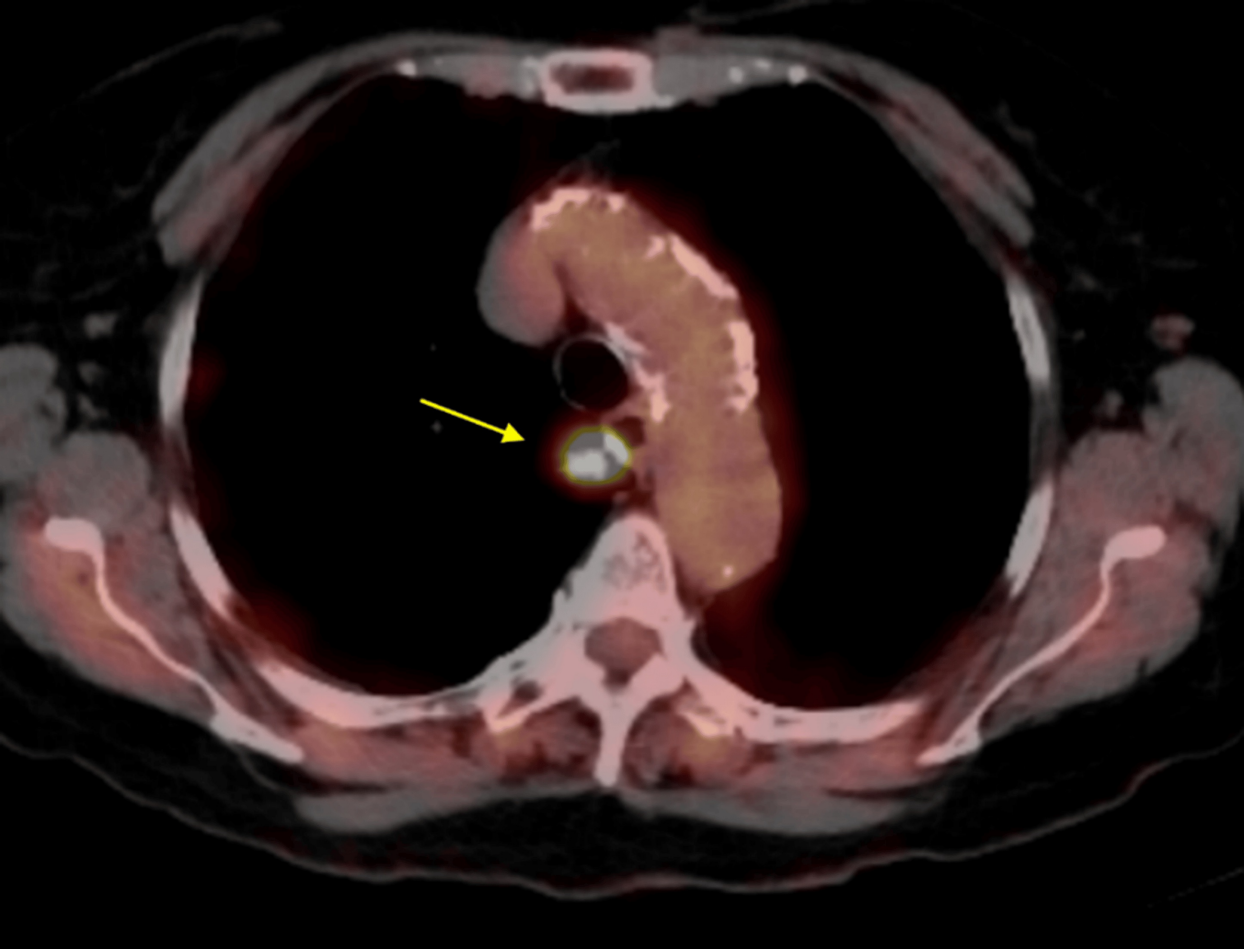
PET scan in November 2024 showing hypermetabolic lesion in the medial portion of the right upper lobe PET: positron emission tomography

The patient underwent a PET scan in November 2024, which also showed the spiculated mass in the medial right upper lobe at the level of the aortic arch, measuring 11 mm. This mass showed a neoplastic degree of hypermetabolism with a maximum SUV of 15 (Figure 7). There was also a small non-calcified nodule in the inferior right upper lobe, measuring 7 mm, which showed no detectable metabolic activity. No other metabolically active lesions were identified. Of note, the PET scan report indicated that the patient would be a poor candidate for CT-guided percutaneous biopsy due to the lesion’s central location and its proximity to the esophagus.

**Figure 8:**
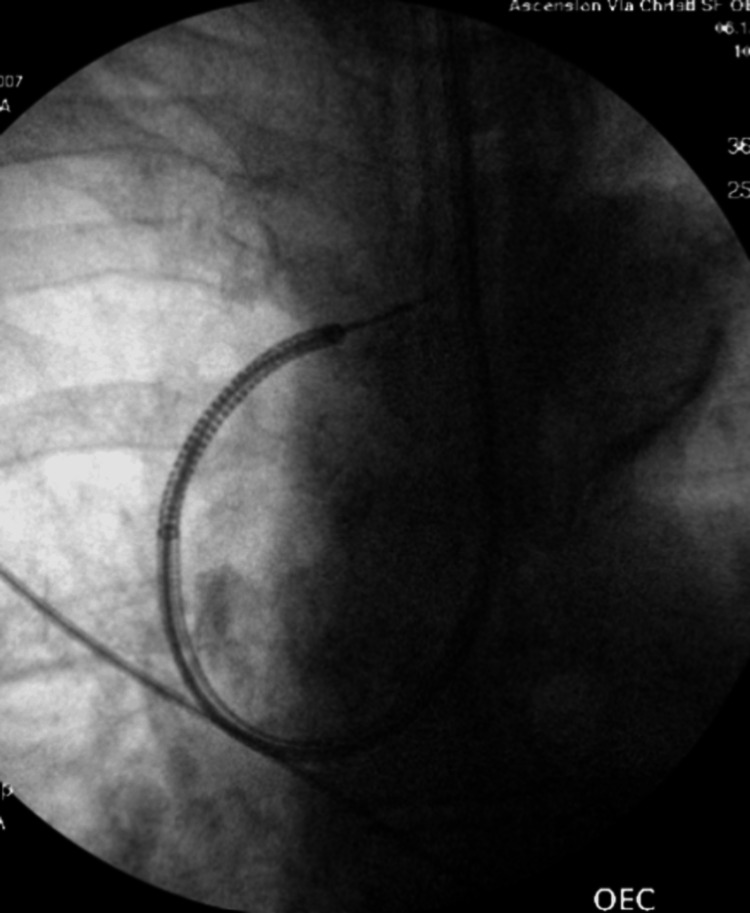
Fluoroscopic image obtained during ION RAB showing the catheter trajectory as it advanced toward the target lesion for biopsy RAB: robotic-assisted bronchoscopy

As a result, the patient underwent an ION RAB with transbronchial biopsy in December 2024 (Figure 8).

On pathology, the morphologic features of the malignant cells favored non-small-cell carcinoma; however, given the overall poorly differentiated nature of the lesion, small-cell carcinoma could not be entirely excluded. The patient was subsequently referred to oncology for further management. She underwent CyberKnife radiotherapy and is currently under active surveillance. Adjuvant chemotherapy was not administered due to her inconclusive biopsy results.

Case 3

**Figure 9:**
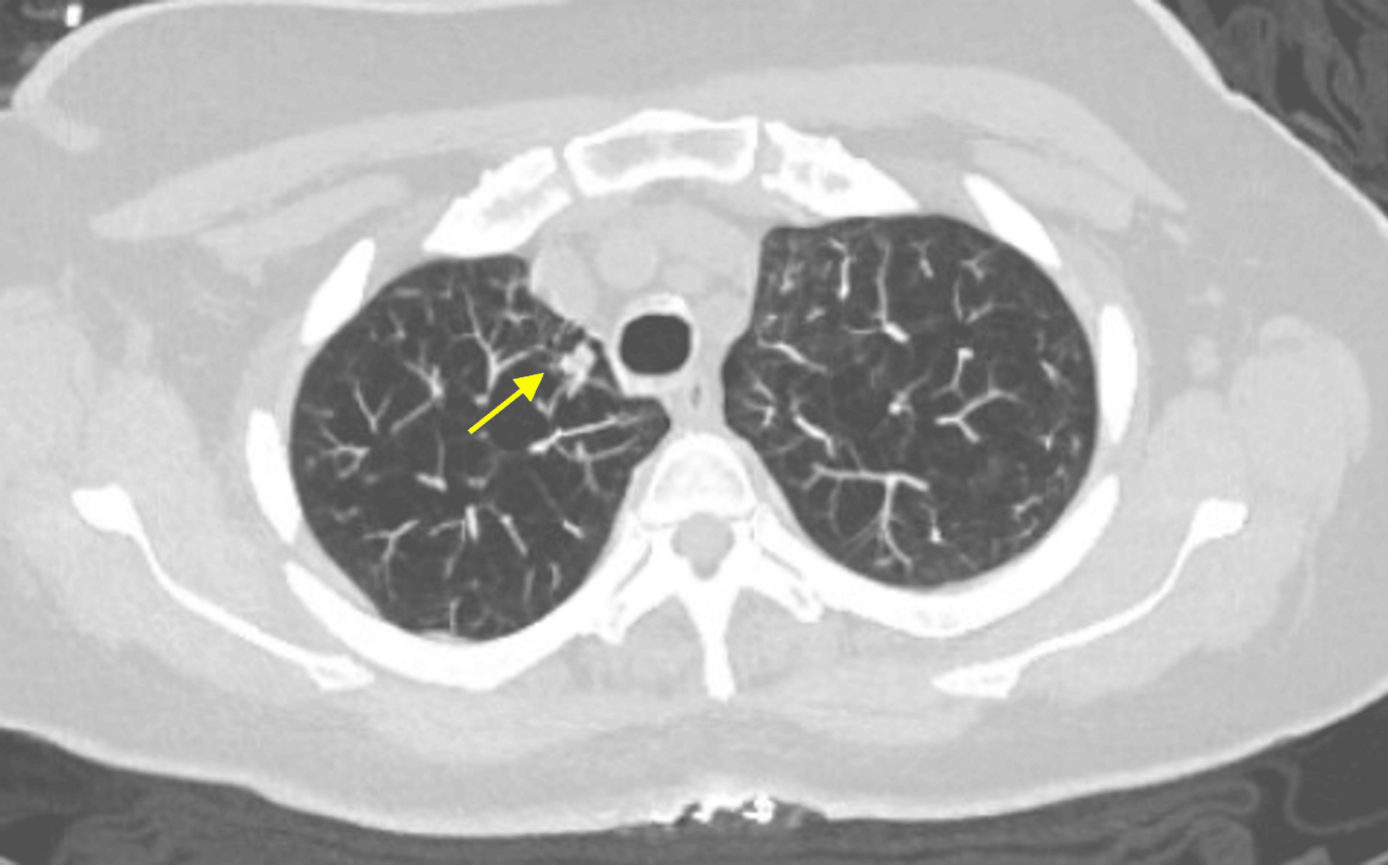
Chest CT scan in June 2024 showing a 7 x 7 mm nodule in the upper lobe of the right lung CT: computed tomography

The patient is a 78-year-old former smoker with COPD. In June 2024, an incidental pulmonary nodule was noted on a CT scan, measuring 7 x 7 mm in the medial right upper lobe (Figure 9).

**Figure 10:**
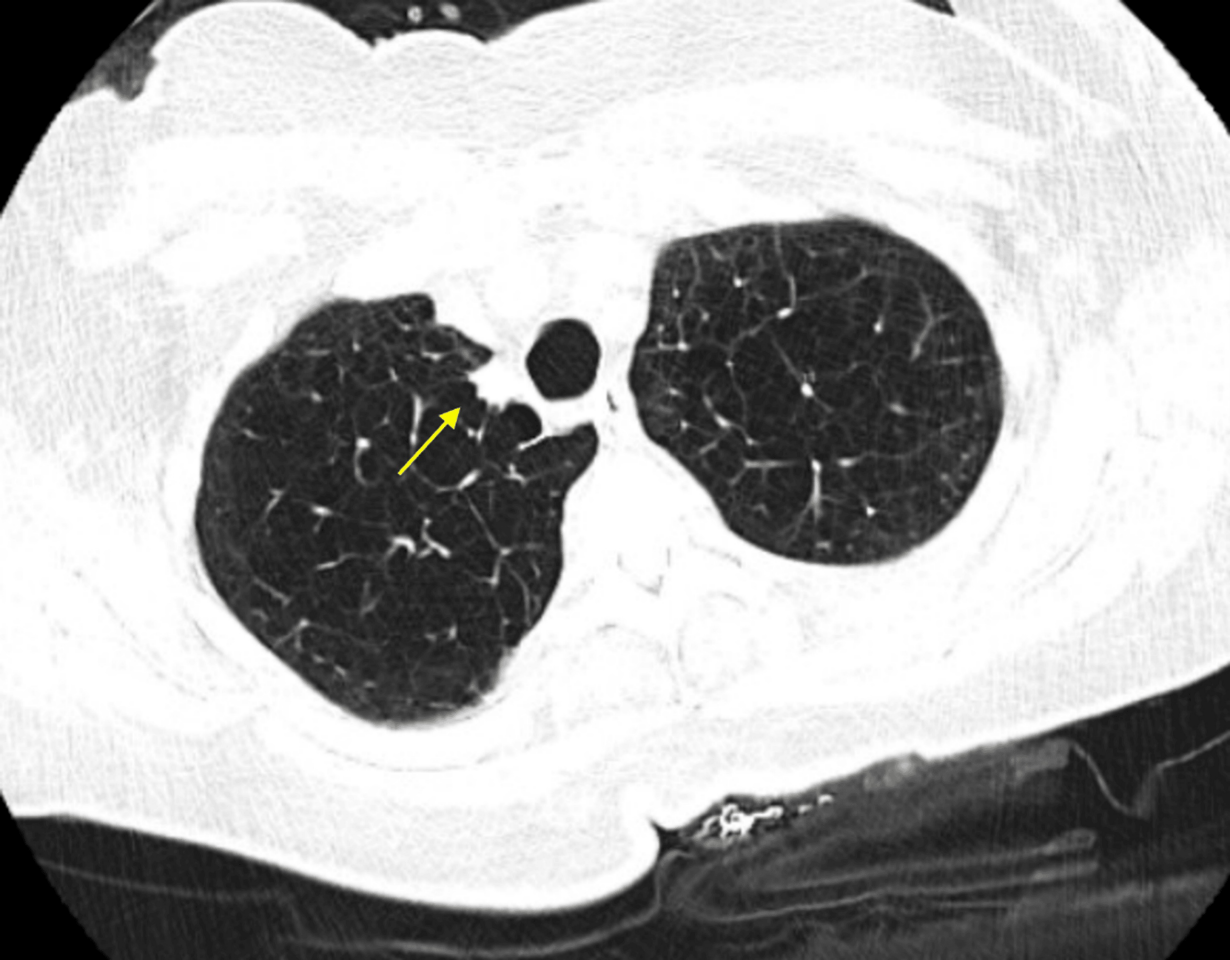
Chest CT scan in September 2024 showing a 10 x 8 mm nodule in the upper lobe of the right lung CT: computed tomography

**Figure 11:**
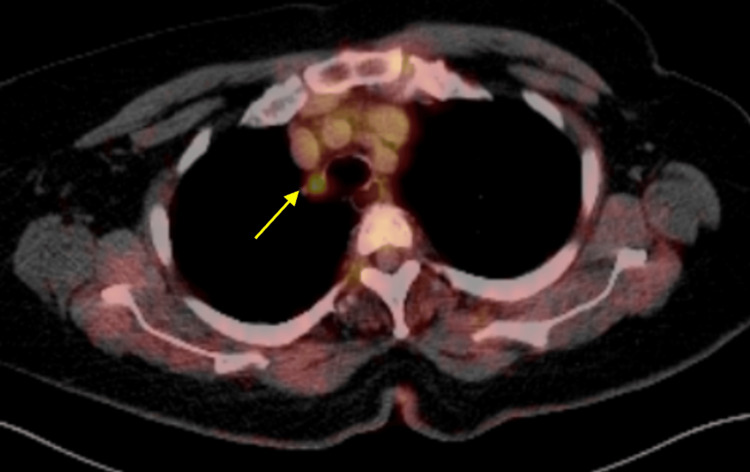
PET scan in November 2024 showing hypermetabolic lesion in the medial portion of the right upper lobe PET: positron emission tomography

**Figure 12:**
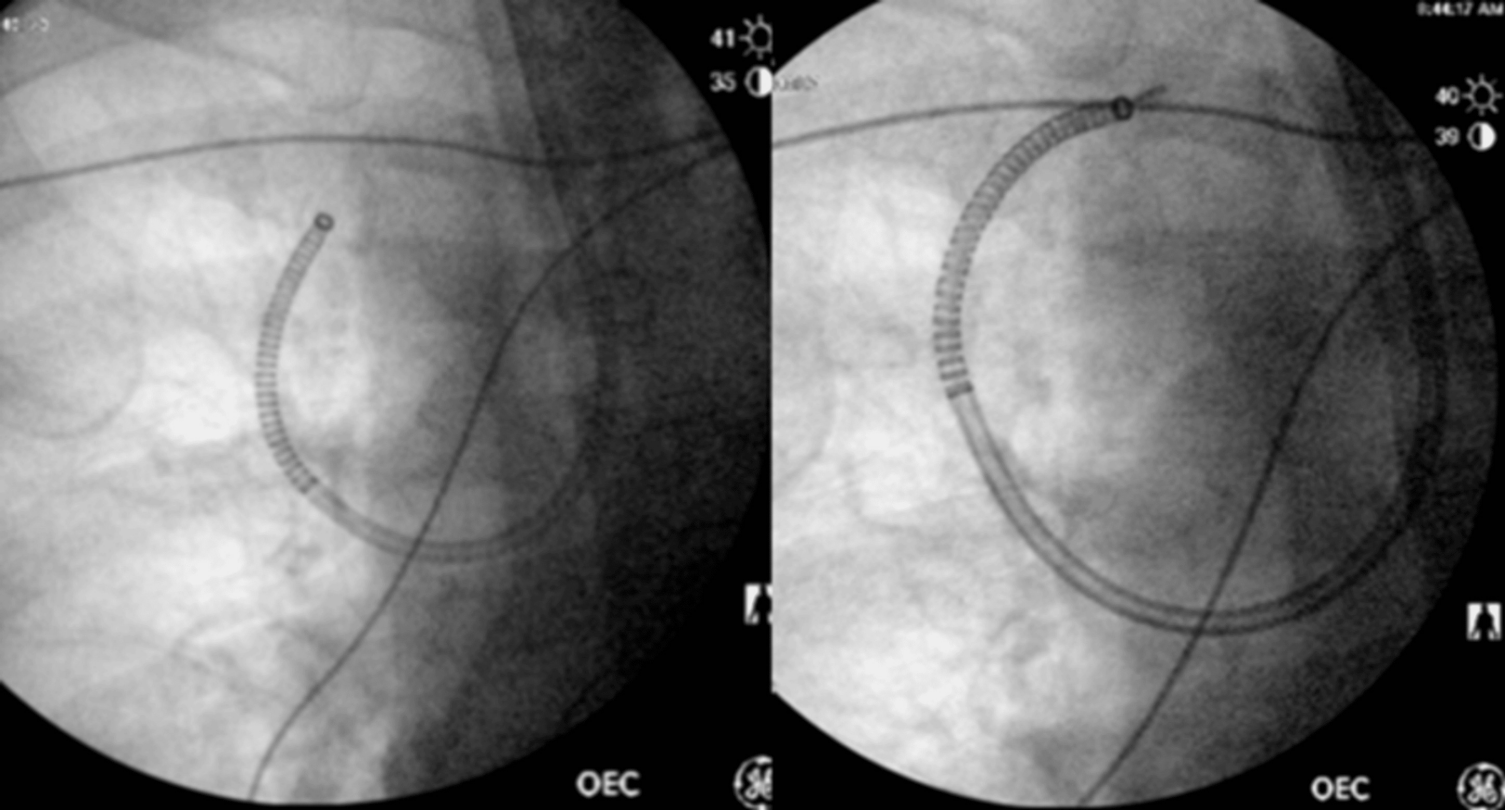
Fluoroscopic images obtained during ION RAB showing the catheter trajectory as it advanced toward the target lesion for biopsy RAB: robotic-assisted bronchoscopy

A follow-up CT scan three months later in September 2024 revealed an increase in the size of the right upper lobe nodule to 10 x 8 mm (Figure 10). A PET scan performed in November 2024 showed a hypermetabolic pulmonary nodule in the medial right upper lobe with a maximum SUV of 5.7 (Figure 11). There were no other areas of hypermetabolic activity suggestive of metastatic disease on the PET scan. As a result, the patient underwent ION RAB with transbronchial biopsy in December 2024 (Figure 12).

Pathology confirmed the presence of small-cell carcinoma. The patient was referred to oncology for stereotactic body radiation therapy. She underwent CyberKnife radiotherapy and received adjuvant chemotherapy with cisplatin and etoposide.

## Discussion

The 2017 Fleischner Society guidelines provide evidence-based recommendations for managing incidentally detected pulmonary nodules in adults ≥35 years old [3]. These guidelines focus on risk stratification, follow-up imaging intervals, and biopsy recommendations based on nodule size, type, and patient risk factors (e.g., smoking history, age, and cancer risk) (Table 1) [3].

**Table 1:** Management and follow-up recommendations for pulmonary nodules according to the 2017 Fleischner Society guidelines CT: computed tomography, PET: positron emission tomography [3]

Nodule type	Size	Initial follow-up	Subsequent follow-up	Comments
Solid nodules	<6 mm (<100 mm³)	No follow-up	No follow-up	Consider CT in 12 months if multiple or high-risk features
Solid nodules	6–8 mm (100–250 mm³)	CT at 6–12 months	Consider 18–24 months if stable	If concern for malignancy, consider PET/CT or biopsy
Solid nodules	>8 mm (>250 mm³)	CT at 3 months, PET/CT, or biopsy	CT at 3 months, PET/CT, or biopsy	Strongly consider tissue diagnosis if suspicious
Multiple solid nodules	<6 mm	No follow-up	CT at 12 months	More concerning if the upper lobe, irregular or increasing
Multiple solid nodules	6–8 mm	CT at 3–6 months	Then 18–24 months if stable	Follow the largest nodule for risk assessment
Pure ground-glass nodule	<6 mm	No follow-up	No follow-up	Malignancy risk is low
Pure ground-glass nodule	≥6 mm	CT at 6–12 months	Then every 2 years for 5 years if persistent	Persistent nodules have a higher malignancy risk
Part-solid nodule	<6 mm	No follow-up	No follow-up	Watch for solid component growth
Part-solid nodule	≥6 mm (solid <6 mm)	CT at 3–6 months	Then annual CT for 5 years	Higher risk than pure ground-glass nodules
Part-solid nodule	≥6 mm (solid ≥6 mm)	Consider biopsy or surgical resection	Consider biopsy or surgical resection	Malignancy risk is high

The National Comprehensive Cancer Network (NCCN) guidelines for managing pulmonary nodules detected during lung cancer screening in the U.S. Preventive Services Task Force eligible patients align with the table above, focusing on risk stratification and appropriate follow-up based on nodule size, density, and patient risk factors (Table 2) [16]. For nodules <6 mm, no follow-up is required [16]. Nodules between 6 and 8 mm should be re-evaluated with a CT scan at 6-12 months and, if stable, with another scan at 18-24 months [16]. Nodules larger than 8 mm require more urgent evaluation, with follow-up CT at three months or, potentially, a PET/CT scan or biopsy [16]. Part-solid and non-solid (ground-glass) nodules ≥6 mm should be closely monitored with longer-term surveillance due to the higher risk of malignancy [16]. These guidelines are designed to optimize early lung cancer detection while minimizing unnecessary interventions, ensuring timely and appropriate management of pulmonary nodules detected through screening [16].

**Table 2:** NCCN 2023 guidelines for pulmonary nodule management (baseline LDCT scan findings) CT: computed tomography, LDCT: low-dose computed tomography, PET: positron emission tomography [16]

Nodule type and size	Follow-up recommendation
No nodule or benign appearance	Annual LDCT
Solid Nodule <6 mm	Annual LDCT
Solid Nodule 6–8 mm	LDCT in 6 months
Solid Nodule 8–15 mm	LDCT in 3 months OR PET/CT
If low suspicion on PET/CT	LDCT in 3 months
If high suspicion on PET/CT	Biopsy or surgical excision
Solid nodule ≥15 mm	Chest CT (± contrast) and/or PET/CT and/or biopsy
If low suspicion	LDCT in 3 months
If high suspicion	Biopsy or surgical excision
Endobronchial nodule	Repeat LDCT in ≤1 month; if persistent, consider bronchoscopy
Part-solid nodule <6 mm	Annual LDCT
Part-solid nodule ≥6 mm, solid component <6 mm	LDCT in 6 months
Part-solid nodule ≥6 mm, solid component 6–8 mm	LDCT in 3 months OR PET/CT
If low suspicion on PET/CT	LDCT in 3 months
If high suspicion on PET/CT	Biopsy or surgical excision
Solid component ≥8 mm	Chest CT (± contrast) and/or PET/CT and/or biopsy
Nonsolid (ground-glass) nodule <20 mm	Annual LDCT
Nonsolid (ground-glass) nodule ≥20 mm	LDCT in 6 months
Multiple nonsolid nodules	Follow dominant nodule size and manage accordingly

In the NLST, the risk of cancer increased with solid lung nodule size: 1.7% for nodules <4 mm, 3.3% for 4 to <6 mm, 5.6% for 6 to <8 mm, 6.5% for 8 to <10 mm, 10.6% for 10 to <15 mm, and 20% for nodules measuring 15-30 mm [17]. Of note, over half of all identified nodules had smooth margins, with 20% spiculated and a quarter poorly defined [17]. For nodules smaller than 10 mm, the cancer rate was significantly higher among those with spiculated margins (7.8%) than among those with smooth margins (3.2%) (p = 0.005) [17]. However, for nodules 10 mm or larger, there was no significant difference in cancer rates between those with spiculated margins (17.6%) and those with smooth margins (23.1%) (p = 0.34) [17].

The data suggest that suspicious pulmonary nodules in patients with significant risk factors for lung cancer have a high likelihood of malignancy. In this high-risk population, the risk of cancer does not appear to differ significantly between nodules measuring 6 to <8 mm and those measuring 8 to <10 mm. This supports the argument that patients with 6 to <8 mm nodules should be managed similarly to those with 8 to <10 mm nodules [17].

Clinical risk can be estimated using several approaches, including validated risk calculators and predictive models. These tools typically consider variables such as age, sex, family history of lung cancer, presence of COPD, and nodule characteristics, including size, location, number, and morphology. Based on these factors, they provide an estimated percentage risk, which can help classify patients into low-, intermediate-, or high-risk categories.

The NCCN and Fleischner Society guidelines use risk stratification to guide the management of solid pulmonary nodules based on patient history and nodule characteristics [3,16]. According to the NCCN, low-risk patients have minimal or no smoking history and lack other risk factors, such as a family history of lung cancer or exposure to environmental toxins like asbestos, radon, or uranium [16]. The FS recommends the risk classification categories proposed by the American College of Chest Physicians, which further define low-risk patients as having an estimated malignancy risk of <5%, with characteristics such as younger age, minimal smoking history, smaller nodules, regular margins, and a non-upper lobe location [3]. In contrast, intermediate (5-65% risk) and high-risk patients (>65% risk) tend to be older, have a history of heavy smoking, larger nodules, irregular or spiculated margins, and upper lobe location. Identifying a patient’s risk level helps determine the appropriate follow-up, balancing early cancer detection with the avoidance of unnecessary procedures [3].

Most malignant lesions typically double in volume every 20 to 300 days, supporting the clinical principle that radiographic stability over two years strongly suggests a benign etiology [2]. This strategy, based on serial CT imaging, is most appropriate when the pretest probability of malignancy is low (<5%), as seen in lung cancer screening trials [2]. However, limitations exist, including delayed diagnosis, potential loss to follow-up, and variability in tumor growth rates. Also, as shown above, the risk of malignancy in high-risk patients with nodules 6-8 mm is >5% [2].

Early diagnosis of lung cancer, particularly SCLC, remains a critical challenge in clinical practice. While current guidelines recommend surveillance for small pulmonary nodules, this strategy may delay diagnosis and treatment, potentially leading to worse outcomes. This paper advocates earlier biopsy in high- and intermediate-risk patients with small pulmonary nodules, especially those smaller than 8 mm. Earlier diagnosis of malignancy may improve the success rate of curative resections and reduce reliance on chemoradiation, which carries significant adverse effects.

Importance of early diagnosis

Earlier detection of pulmonary malignancies, particularly those arising in high-risk patients, can dramatically improve patient outcomes. For patients with small nodules, waiting for several rounds of follow-up imaging may allow the tumor to grow and spread, ultimately reducing the opportunity for curative treatment. A biopsy performed earlier in the process would enable timely identification of malignancy, ensuring patients receive appropriate treatment as early as possible. This could lead to fewer patients requiring more aggressive treatments, such as chemoradiation, which often have significant side effects and are generally associated with poorer prognoses.

Limitations of current surveillance guidelines

Current guidelines, such as those from the NCCN and Fleischner Society, primarily focus on surveillance with repeat imaging at intervals, depending on nodule size and risk factors. However, waiting for follow-up scans before taking further action may not be ideal for all patients, particularly those in high-risk groups. High- and intermediate-risk patients with smaller nodules may experience significant growth during the waiting period, which may reduce the chances of successful curative treatment [3]. Additionally, the stress and anxiety of waiting for follow-up imaging can take a toll on the patient’s mental health and may contribute to loss to follow-up [18]. Earlier biopsies could prevent unnecessary delays in diagnosis and enable the prompt initiation of appropriate treatments.

Impact of early biopsy on management and treatment

An earlier biopsy of smaller pulmonary nodules in high-risk patients could significantly change the course of management. In cases where malignancy is diagnosed promptly, surgical resection could be performed before the tumor has a chance to metastasize, increasing the likelihood of a curative outcome and reducing the risk of recurrence [10]. Early diagnosis would allow clinicians to make more informed treatment decisions, potentially avoiding the need for primary chemoradiation later. This not only improves the patient's quality of life by minimizing the long-term side effects of more invasive therapies but also increases overall survival by enabling timely intervention.

Impact of delayed diagnosis

As demonstrated in the cases presented, high-risk patients with small pulmonary nodules can show significant growth while awaiting follow-up imaging. In these cases, waiting for follow-up scans delayed crucial diagnosis and, in some instances, led to worse outcomes as the nodules had grown significantly by the time they were re-imaged. These examples underscore the potential consequences of waiting too long before taking action. Early biopsies could prevent this delay, ensuring that high-risk patients with suspicious nodules receive the attention and care they need right from the start.

SCLC and the potential for earlier diagnosis

SCLC, which is notoriously aggressive and characterized by rapid metastasis, represents a challenge in lung cancer diagnosis and management [19]. Due to the nature of the disease, surgery is typically not an option, as only very few patients are diagnosed with stage 1 disease, where surgery is an option [19]. However, earlier biopsy of suspicious pulmonary nodules, even those smaller than 8 mm, could potentially lead to earlier identification of SCLC. A tissue diagnosis at this stage could allow for a more tailored approach to treatment, possibly enabling more effective interventions before the cancer advances. Given that SCLC has a low rate of Stage I diagnosis due to its rapid growth, earlier biopsies could enhance the detection of more resectable or treatable cases, ultimately improving survival outcomes and quality of life for patients [19].

Role of robotic-assisted bronchoscopy

The ION RAB platform exemplifies the technological advancements that support the central argument of this paper: earlier biopsy of smaller pulmonary nodules, especially in intermediate- or high-risk patients, can and should be pursued when appropriate tools are available. ION’s precision and reach across all lung segments enable safe, accurate sampling of nodules previously deemed too small or inaccessible for biopsy [14,15]. By minimizing procedural risk and maximizing diagnostic yield, ION RAB reinforces the rationale for shifting toward earlier intervention, potentially enabling earlier detection of malignancy and improving clinical outcomes through timely treatment [11,12]. RAB enables precise navigation to peripheral and distal lung nodules and can be combined with EBUS and cone-beam CT for real-time localization and tool confirmation, enhancing diagnostic yield and procedural control compared with CT-guided cone-beam radial EBUS alone [14,15].

## Conclusions

The case for earlier biopsy of small pulmonary nodules in high- and intermediate-risk patients is compelling. Delaying tissue diagnosis through repeated imaging not only risks missing the window for curative resection but may also lead to the unnecessary use of chemoradiation, which comes with its own set of significant side effects. By embracing earlier biopsies for smaller nodules, especially those in high-risk populations, clinicians could increase the likelihood of early malignancy detection, improve patient outcomes, and reduce the need for aggressive treatments. Incorporating advanced technologies like ION RAB makes this shift toward earlier biopsy both feasible and safe, offering precise access to small, peripheral nodules with high diagnostic yield and low complication rates. The current approach of waiting for follow-up imaging warrants further evaluation, particularly in high-risk individuals. Prospective studies or registries are needed to determine whether earlier intervention could safely improve outcomes and inform optimal management strategies in lung cancer patients.
